# The dual roles of chemokine (C-C motif) ligand 7 in tumors and inflammatory diseases

**DOI:** 10.7717/peerj.20685

**Published:** 2026-01-29

**Authors:** Danhong Qian, Yikang Mo, Jinji Chen, Wenqing Guan, Lihui Yan, Qi Zhou

**Affiliations:** Hangzhou Linping District Integrated Traditional Chinese and Western Medicine Hospital, Hangzhou, China

**Keywords:** CCL7, Tumor, Inflammation, Chemokine

## Abstract

Chemokines are regarded as major contributors to tumor growth and inflammation. They participate in cancer progression directly and accelerate tumor microenvironment (TME) remodeling because of the inflammatory response and immune cell infiltration. C-C motif chemokine ligand 7 (CCL7) is widely expressed across various cell types. In tumors, CCL7 facilitates progression by shaping the TME and promoting cell invasion and metastasis. It also enhances anti-tumor immune responses by recruiting immune cells such as T cells and NK cells. Moreover, emerging evidence indicates that the overexpression of CCL7 is associated with inflammatory diseases. CCL7 predominantly attracts macrophages and monocytes, thereby modulating inflammatory responses, driving fibrosis progression, and maintaining systemic homeostasis. However, the exact mechanisms underlying these processes remain unclear and lack systematic investigation. This review systematically integrates CCL7’s dual roles in tumors (both pro-tumor and anti-tumor) and its stage-specific functions across inflammation (both pro-inflammatory and anti-inflammatory), fibrosis, and obesity, filling gaps in fragmented prior research. It also links the common regulatory mechanisms across multiple diseases, and puts forward specific strategies such as CCL7/CCR antagonists and combined immunotherapy, thereby providing new therapeutic strategies for the exploration and treatment of related diseases, aiming to contribute to the maintenance of human health globally.

## Introduction

Chemokines are small proteins (8–12 kDa) initially characterized by their ability to attract various immune cells, such as monocytes, lymphocytes, and neutrophils, to sites of tissue injury and infection. Through this process, they play a pro-inflammatory role while mediating immune responses (Braoudaki et al., 2022; Mantovani et al., 2022). Beyond their established immunological functions, recent studies have revealed their involvement in tumor progression and the maintenance of metabolic homeostasis. Based on the number and arrangement of conserved cysteine residues, chemokines are classified into four families: CXC, CC, C, and CX3C. Their diverse functions depend on interactions with receptors from the CCR family, which are expressed on the surface of target cells (Braoudaki et al., 2022; La Cognata et al., 2024).

CCL7, also known as monocyte chemoattractant protein 3 (MCP-3), is a member of the CC subfamily and was first identified in the supernatant of osteosarcoma cells (Van Damme et al., 1992). Under physiological and pathological contexts, CCL7 is released by diverse cellular sources, physiologically, it is secreted by endothelial cells and leukocytes; pathologically, it is produced by tumor cells and cancer-associated fibroblasts (CAFs). The expression of CCL7 is regulated by pro-inflammatory cytokines (such as TNF-α, IL-1β) and hypoxic microenvironmental cues (Chang et al., 2024). CCL7 exhibits bidirectional regulatory roles in orchestrating inflammatory responses, modulating tumor immunity, mediating tissue repair, and governing disease progression: on one hand, it reinforces anti-tumor immune surveillance (LeBleu & Kalluri, 2018), on the other hand, it potentiates tumor malignant invasion and metastasis by driving cancer cell migration and EMT *via* autocrine and paracrine signaling loops (Korbecki et al., 2020).

Moreover, the aberrant elevation of CCL7 has been reported in other diseases, including psoriatic skin lesions (Brunner et al., 2015), HIV infection (Atluri et al., 2016), acute neutrophilic pulmonary inflammation (Choi et al., 2004), and pulmonary fibrosis (Liu et al., 2023). These findings underscore the pivotal role of CCL7 in both tumors and various immune-related disorders. In this review, we summarize the diverse functions of CCL7 in tumorigenesis and immune-mediated diseases and aim to provide new insights that may inform therapeutic strategies for related conditions. Finally, the review emphasizes the effect of chemokines in TME and inflammation, making it a valuable resource for scientists working at the intersection of inflammatory diseases, anti-tumor treatment and metabolic disorders.

## Survey Methodology

The review conducted a large amount of literature articles on PubMed, using search terms such as CCL7, tumor, inflammation, fibrosis, obesity and macrophages. A comprehensive publications search was performed on the PubMed database, covering publications from database inception to March 2025. The search strategy combined MeSH Terms and free text words, with the following logical combination: (“CCL7” OR “Chemokine C-C Motif Ligand 7” OR “MCP-3” OR “Monocyte Chemoattractant Protein 3”) AND (“tumor” OR “cancer”) AND (“inflammation”) AND (“fibrosis”) AND (“obesity”) AND (“macrophages”). Two independent researchers (Danhong Qian and Qi Zhou) performed the search separately, with discrepancies resolved through discussion.

For inclusion, studies were selected based on two core criteria: first, content relevance, we considered studies only explicitly explored CCL7’s biological functions (such as CCL7-mediated cell migration and signaling activation), regulatory mechanisms, or clinical implications in the context of tumors, inflammatory diseases, fibrosis, obesity, or macrophages. Second, study type included publications encompassed original research articles, meta-analyses and systematic reviews with clear literature screening protocols and data synthesis methods, and randomized controlled trials that focus on CCL7-targeted interventions. Studies were excluded based on two key factors: in terms of study type, letters to the editor, case reports, commentaries, editorials, conference abstracts, and unpublished manuscripts were excluded due to their limited data, lack of systematic design, or unvalidated results; regarding content relevance, studies that only mentioned CCL7 in the background or discussion sections without exploring its specific role in the aforementioned target diseases were also excluded. In total, 103 publications were cited in this review.

## Structure and Receptors of CCL7

### The structure of CCL7

The human CCL7 gene is located on chromosome 17q11.2-12 and comprises three exons and two introns. The first exon contains the 5′-UTR, an N-terminal signal sequence (23 amino acids), and the first two amino acids of the mature protein. The second exon encodes amino acids 3 to 42 of the mature protein, while the final exon encodes the C-terminal region and the 3′-UTR, which includes one or more AU-rich instability elements and a eukaryotic polyadenylation signal (Opdenakker et al., 1994; Weber et al., 1999). The transcription start site lies 16 base pairs downstream of the TATA box in the promoter region. Several regulatory elements modulate transcription upstream of the promoter: CRE and Ets-like elements notably suppress promoter activity (Opdenakker et al., 1994). Two enhancers are present—one spanning positions −172 to −110 and another located at position −37, only 21 base pairs upstream of the TATA box, known as the AP-1-like element (Murakami et al., 1997).

### The receptor CCRs of CCL7

The CC chemokine receptors CCR1, CCR2, and CCR3 are widely recognized as the principal functional receptors of CCL7 (Griffith, Sokol & Luster, 2014; Palomino & Marti, 2015; Lee et al., 2016). Additionally, CCL7 has been reported to bind CCR5 (Jung et al., 2010) and CCR10 (Ford et al., 2019). These small chemokine receptors have a short N-terminal region and a short third intracellular loop. The C-terminal region is rich in serine and threonine residues, which are phosphorylation sites upon receptor activation. The extracellular loops and N-terminal region are essential for ligand binding (Murphy, 1996). The interaction of CCL7 with different receptors mediates distinct biological functions.

CCL7 is the ligand to CCR1, which is expressed in various cells such as monocytes, T cells, dendritic cells and neutrophils. Its protein consists of 355 amino acid residues and belongs to the peptide subfamily of the Class A G protein-coupled receptor family (Cheng & Jack, 2008). CCR1 signaling may contribute to inflammation by binding to CCL7 and facilitating macrophage infiltration (Wei et al., 2024). CCR2 is a widely studied and functional receptor expressed on the surface of monocytes/macrophages and lymphocytes, controlling monocyte mobilization, infiltration and their migration from the bloodstream to injured organs and damaged tissues. In colorectal cancer patients, elevated expression of both CCR2 and CCL7 has been observed, with overexpression of CCL7 significantly correlating with poorer survival outcomes (Kurzejamska et al., 2019). In the liver, damaged hepatocytes or hepatic stellate cells can promote macrophage infiltration, thus leading to inflammation and fibrosis by producing CCL7 (She et al., 2022). The CCL7-CCR3 axis regulates bone metabolism by promoting osteoblast differentiation and remodeling, offering potential therapeutic avenues for osteoporosis (Kim et al., 2024). Furthermore, the CCL7-CCR5 interaction is key in neuropsychiatric disorders and pain modulation (Szabo et al., 2002).

The expression profiles of chemokine receptors are highly complex and context-dependent, influenced by factors such as cell lineage, differentiation state, and microenvironmental cues—including chemokine concentration, presence of inflammatory cytokines, and hypoxia (Laurent et al., 2016).

## The Role of CCL7 in Tumors

**Figure 1:**
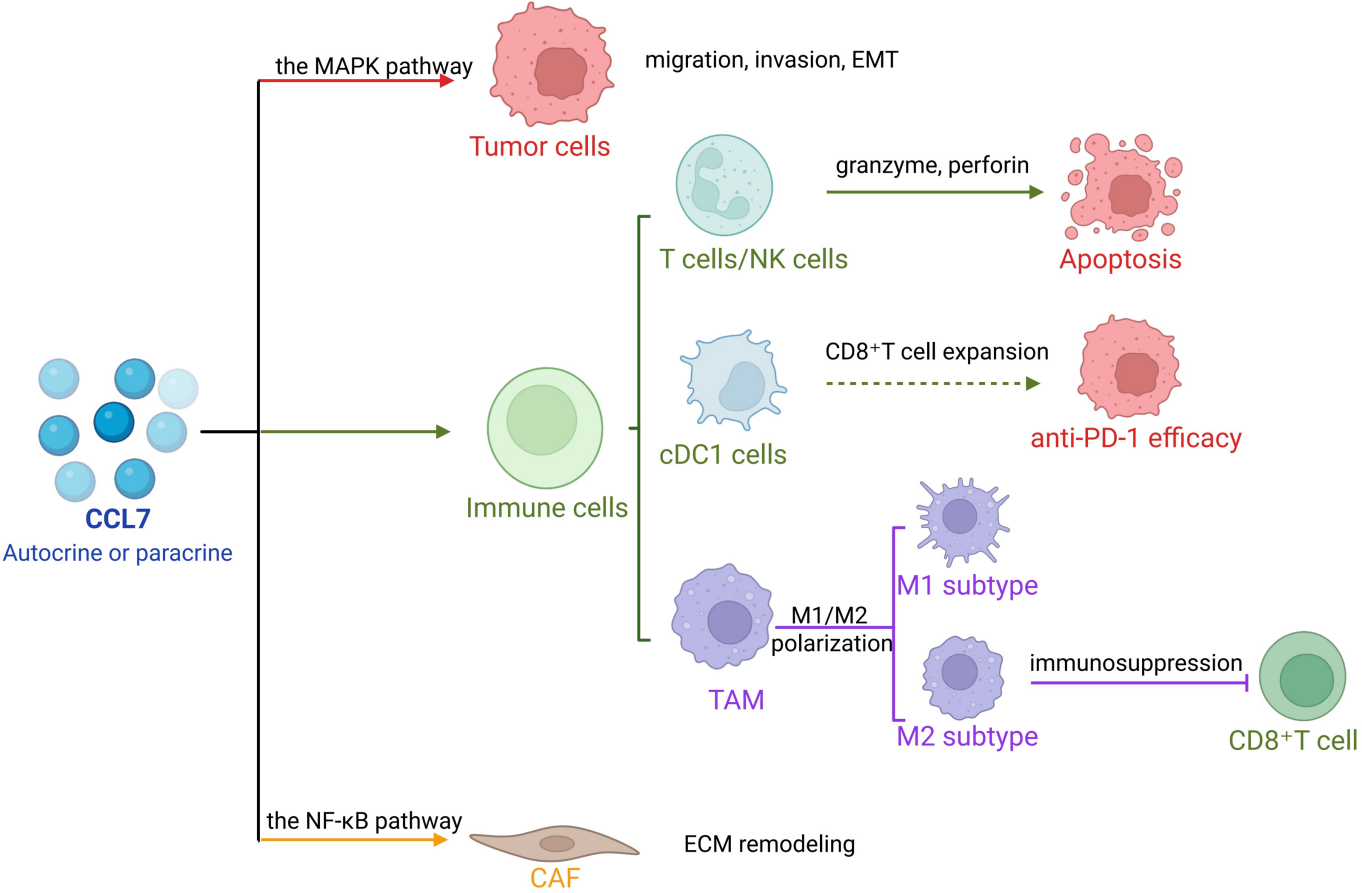
The role of CCL7 in tumors.

CCL7 plays a pivotal role in tumorigenesis and tumor progression (Liu et al., 2018). In addition to intrinsic alterations within tumor cells, cancer development is profoundly influenced by interactions between tumor cells and stromal components within TME, which include fibroblasts, macrophages, and adipocytes (Barreirada Silva et al., 2022; Kline et al., 2024; Su et al., 2023). As a key molecular mediator in this crosstalk, CCL7 drives tumor progression and, under certain conditions, may also exert anti-tumor effects (Fig. 1).

### Pro-tumor effects of CCL7

CCL7 promotes tumor growth, migration, and invasion. Autocrine signaling of CCL7 enhances tumor cell proliferation; in colorectal cancer (CRC) cells, lentiviral overexpression of CCL7 facilitates its binding to CCR3, activating the downstream Extracellular Regulated Protein Kinases (ERK)/ c-Jun N-terminal Kinases (JNK) signaling cascade of the mitogen-activated protein kinase (MAPK) pathway. This activation promotes epithelial-mesenchymal transition (EMT) and enhances the metastatic potential of CRC cells (Lee et al., 2016). Furthermore, CCL7 secreted by surrounding stromal cells contributes to tumor progression. In lung adenocarcinoma, CCL7 modulates immune responses within the tumor microenvironment, enhancing invasiveness (Parikh et al., 2018). Overexpression of SOX18—a critical indicator of survival and recurrence in gastric cancer—positively correlates with CCL7 levels, with CCL7 serving as a direct transcriptional target of SOX18. The CCL7-CCR1-SOX18 axis forms a positive feedback loop that continuously promotes gastric cancer invasion and metastasis (Chen et al., 2020b).

### Anti-tumor effects of CCL7

As a chemokine, CCL7 recruits various leukocyte subsets, triggering immune responses. In early tumor development, fibroblasts can recruit and activate immune cells to generate extracellular matrix components that suppress tumor growth (LeBleu & Kalluri, 2018). In response to CCL7 activation, T lymphocytes enhance the production of granzyme and perforin, mediating tumor cell apoptosis. Moreover, CCL7 has been shown to inhibit tumor growth in a concentration-dependent manner. For instance, in the presence of CCL7, B78/H1 melanoma cells fail to establish tumors in murine models, and K1735 melanoma shows significantly reduced tumor formation and growth (Wetzel et al., 2007). Similarly, Infection of HeLa cells with hH1/MCP-3 induces high MCP-3 secretion, markedly suppressing tumor growth in recipient mice compared to controls (Hu et al., 2002).

In non-small cell lung cancer (NSCLC), CCL7 recruits conventional dendritic cell type 1 (cDC1), promotes CD8^+^ T cell expansion, and enhances the efficacy of anti-PD-1 therapy. CCL7 alone or in combination with PD-1 blockade significantly inhibits tumor growth (Zhang et al., 2020). In ovarian cancer, CCL7 deficiency accelerates tumor growth and reduces sensitivity to PD-1 inhibitors (Mollaoglu et al., 2024). Cancer cells may co-opt fibroblasts as fibrosis progresses, shifting their function from tumor suppression to tumor promotion (Kalluri, 2016; Hanahan & Weinberg, 2011). Thus, CCL7 holds promise as a novel molecular target for anti-tumor therapy (Table 1).

**Table 1:** The role of CCL7 in tumors.

Effect type	Core mechanism	Involved diseases/models	Key molecules/cells	Related receptors/signaling pathways	References
Pro-Tumor Effects	Promotes tumor cell proliferation, migration, and invasion	Colorectal Cancer	CCL7, ERK, JNK, EMT-related molecules	CCR3, MAPK	Lee et al. (2016)
	Regulates the Tumor Microenvironment (TME) and enhances invasiveness	Lung Adenocarcinoma	CCL7, TME immune cells	Not specified	Parikh et al. (2018)
	Forms a positive feedback loop to drive metastasis	Gastric Cancer	CCL7, SOX18	CCR1	Chen et al. (2020b)
	Enhances tumor angiogenesis and immunosuppression	Various solid tumors	CCL7, TAMs, VEGF, TGF-β, T cells	CCR1/CCR2/CCR3	Kurzejamska et al. (2019), Yang & Zhang (2017) and Mohr et al. (2017)
	Activates CAFs and remodels the TME	Oral Squamous Cell Carcinoma, Hepatocellular Carcinoma, Breast Cancer	CCL7, CAFs, NF-κB, TGF-β, Smad2	CCR2; NF-κB, TGF-β/Smad2	Sahai et al. (2020), Jung et al. (2010), Huang et al. (2024) and Pestell et al. (2017)
	Promotes the formation of a liver metastatic microenvironment	Colorectal Cancer Liver Metastasis	CCL7, HIF-1α, ERK	ERK	Chang et al. (2024) and Mohr et al. (2017)
Anti-Tumor Effects	Recruits effector immune cells and activates anti-tumor immunity	Melanoma	CCL7, T lymphocytes, NK cells,	Not specified	Wetzel et al. (2007)
	Inhibits tumor growth	Colorectal Cancer	CCL7, hH1/MCP-3	Not specified	Hu et al. (2002)
	Enhances the efficacy of immune checkpoint therapy	Non-Small Cell Lung Cancer (NSCLC)	CCL7, cDC1, CD8^+^T cells, PD-1	Not specified	Zhang et al. (2020)
	Maintains sensitivity to PD-1 inhibitors	Ovarian Cancer	CCL7, TAMs, PD-1	Not specified	Mollaoglu et al. (2024)

### CCL7 and TME

As cancer research advances, increasing attention is being directed toward the TME, the dynamic ecosystem surrounding tumor cells, which includes vasculature, extracellular matrix (ECM), diverse signaling molecules, immune cells, fibroblasts, adipocytes, and other non-malignant components (Giraldo et al., 2019).

### Tumor-associated macrophages

Tumor-associated macrophages (TAMs), predominantly composed of M2 macrophages with a minor M1 component, are implicated in tumor initiation, progression, angiogenesis, and metastasis (Mantovani et al., 2017; Xiang et al., 2021).

CCL7 plays a crucial regulatory role within the TME by modulating macrophage behavior. For T cells, TAMs foster angiogenesis and suppress T cell-mediated anti-tumor responses, thereby facilitating tumor progression (Yang & Zhang, 2017). In contrast, CCL7 downregulation enhances anti-tumor immunity *via* two key mechanisms: boosting CD8^+^ T cell infiltration and reversing T cell exhaustion (Zhang et al., 2025). Beyond this, elevated CCL7 levels create a favorable microenvironment for colorectal cancer hepatic metastasis (Mohr et al., 2017). In the liver, resident macrophages (Kupffer cells, KCs) modulate ECM remodeling to alter the hepatic tumor microenvironment (Kang, Gores & Shah, 2011; Rasool et al., 2013). Additionally, CCL7 secreted by immature monocytes or neutrophils enhances the migration and invasion of NSCLC cells (Zhang et al., 2020).

### CCL7 and CAFs

CAFs are key players in tumor progression and represent a significant focus in oncology research (Zhang et al., 2023; Sahai et al., 2020). Typically derived from quiescent fibroblasts, CAFs drive tumor progression *via* CCL7-mediated intercellular crosstalk and downstream mechanisms (Sahai et al., 2020; Chen, McAndrews & Kalluri, 2021; Biffi & Tuveson, 2020).

First, CAFs could directly secret growth factors to promote tumor proliferation and metastasis. Activation of the nuclear factor kappa-B (NF-κB) pathway in CAFs significantly upregulates CCL7 secretion, promoting tumorigenesis. Conversely, suppression of CCL7 expression markedly impairs tumor growth (Jung et al., 2010). In hepatocellular carcinoma, elevated CCL7 expression activates the TGF-β/Smad2 signaling pathway to enhance tumor cell migration and invasion (Huang et al., 2025). Secondly, CAFs indirectly support tumor development through TME remodeling. CCL7 stimulation induces CAFs to secrete ECM-modulating factors, strengthen their interactions with tumor cells and facilitate tumor migration (Pestell et al., 2017). Collectively, these findings highlight the multifaceted role of CCL7 in modulating TME and driving tumor progression *via* diverse molecular pathways.

### Targeting CCL7-CAFs axis for anti-tumor therapy

In terms of checkpoint inhibitors, in NSCLC, CCL7 recruits cDC1 to enhance CD8^+^ T cell expansion, thereby potentiating anti-PD-1 efficacy (Zhang et al., 2020). In contrast, CCL7 plays a role in maintaining sensitivity to PD-1 inhibitors in ovarian cancer. CCL7 deficiency reduces TAM infiltration and exacerbates anti-PD-1 resistance, while CCL7 expression reverses this phenotype (Mollaoglu et al., 2024). These findings support CCL7 as a predictive biomarker for checkpoint inhibitor response (Zhang et al., 2020; Mollaoglu et al., 2024). For CCL7-CAFs strategies, CAFs could directly secrete CCL7 through the NF-κB pathway (Jung et al., 2010), and CCL7 drives tumor progression *via* activation of the TGF-β/Smad2 (Huang et al., 2025) pathway. It also induces CAFs to secrete ECM-modulating factors such as collagens to create a microenvironment conducive to tumor metastasis (Pestell et al., 2017). Thus, targeting CCL7 could simultaneously inhibit both the direct pro-tumor effects and TME remodeling functions of CAFs. Therefore, targeting the key pathways that regulate CCL7 expression in CAFs, blocking their upstream activation signals *via* small-molecule inhibitors to reduce CCL7 production is feasible, such as NF-κB inhibitors (BAY 11-7082). Additionally, interfering with the interaction between CCL7 and its receptors (CCRs) using antibodies or antagonists, or inhibiting the activation of its downstream pathways, can disrupt the regulatory effects of CAF-derived CCL7 on tumor cells.

## The Role of CCL7 in Inflammation-related Diseases

Chemokines can direct monocytes and macrophages circulating within the bloodstream to various sites, where they orchestrate inflammatory responses and help maintain homeostasis (Mutsaers et al., 2023; Coussens & Werb, 2002). Several kinds of immune cells are involved in the occurrence and progression of inflammation and fibrosis caused by CCL7. Here, we put more emphasis on macrophages and their function in inflammation, fibrosis and glucose metabolism (Table 2).

**Table 2:** The role of CCL7 in inflammation-related diseases.

Effect type	Disease/Context	Affected cells	Key mechanisms	CCL7′s role performance	References
Pro-inflammatory	Liver injury	Hepatocytes, monocytes/macrophages	Hepatocytes secrete CCL7 to recruit inflammatory cells; CCL7 deficiency alleviates damage.	Exacerbates liver inflammation	Kong et al. (2021)
	Psoriatic skin lesions	Monocytes/ macrophages	TNF-α/IL-1β drives CCL7 upregulation; recruits macrophages to worsen lesions.	Aggravates skin inflammation	Brunner et al. (2015)
	IBD	Pro-inflammatory macrophages	Persistent CCL7-CCR2 activation recruits pro-inflammatory macrophages.	Sustains chronic intestinal inflammation	He et al. (2019)
	Allergic airways disease	Macrophages, neutrophils	Recruits inflammatory cells; activates NF-κB pathway to amplify inflammation.	Worsens lung allergic inflammation	Girkin et al. (2015)
	Osteoarthritis (Inflammatory Phase)	M1 macrophages	Promotes M1 macrophage polarization and inflammatory factor secretion.	Aggravates early joint inflammation	Xu et al. (2025)
	Obesity	Adipocytes, M1 macrophages	Adipocytes upregulate CCL7; binds CCR5 to enhance adipose inflammation.	Enhances adipose tissue inflammation	Bou Malhab et al. (2024) and Huber et al. (2008)
	Acute pulmonary inflammation	Monocytes, neutrophils	Elevated CCL7 recruits inflammatory cells to lungs.	Exacerbates acute lung inflammation	Choi et al. (2004)
Anti-inflammatory	Cutaneous Leishmania Infection	Neutrophils, monocytes	Antagonizes neutrophil migration; stabilizes monocyte distribution.	Inhibits excessive skin inflammation	Ford et al. (2019)
	Osteoarthritis	Monocytes	Binds CCR to stabilize monocyte distribution; reduces joint inflammatory cell infiltration.	Alleviates joint pain/inflammation	Pandey et al. (2024)
	Systemic inflammation	Circulating/bone marrow monocytes	Maintains monocyte homeostasis; restricts systemic hyperinflammation.	Suppresses systemic inflammation	Tsou et al. (2007)

### Inflammation-associated monocytes/macrophages

Monocytes are recruited to inflamed tissues, where they differentiate into macrophages capable of synthesizing a broad array of cytokines and growth factors, such as TGF-β and matrix metalloproteinases (MMPs), which are pivotal to host defense responses. Macrophages can be polarized into M1/M2 phenotypes with distinct functions. M1 macrophages are typically linked to pro-inflammatory responses with secretion of cytokines like TNF-α, IL-6 and IL-1β, while M2 macrophages are correlated with anti-inflammatory effects, secreting IL-4 and IL-10 (Varol, Mildner & Jung, 2015). However, macrophages exhibit high plasticity for M1/M2 polarization could dynamically shift in response to the surrounding microenvironment. A single macrophage population could switch between M1 and M2 phenotypes in response to different signals and diverse stimulation both *in vivo* and *in vitro*. Therefore, when we analyze how CCL7 affects macrophage polarization, it is necessary to consider carefully in the context of specific physiological scenarios, tissue types and species differences, instead of limiting our understanding of macrophages to the traditional M1/M2 classification framework.

The dual role of CCL7 in macrophage polarization is complex. On one hand, CCL7 could promote macrophage migration and exacerbate inflammatory processes. During liver injury, hepatocyte-derived CCL7 enhances inflammatory cell infiltration. CCL7 is highly increased in lesional psoriatic skin by TNF-a/IL-1b axis in mice (Brunner et al., 2015). In CCL7-deficient mice, both LPS- and MCD-induced liver injuries are mitigated, underscoring CCL7’s modulatory role in acute and chronic liver damage (Kong et al., 2021). In patients with inflammatory bowel disease, elevated CCL7-CCR2 signaling correlates strongly with macrophage infiltration, with heightened CCL7 expression driving the accumulation of pro-inflammatory macrophages in the intestinal tract (He et al., 2019).

On the other hand, neutrophils are more easily infiltrated into the infected skin to cause inflammation in CCL7-deficient mice, and improving the expression of CCL7 *in vivo* could alleviate inflammatory infections. *In vitro*, CCL7 can also directly antagonize neutrophil migration (Ford et al., 2019). CCL7 exerts anti-inflammatory and analgesic effects by binding to CCR receptors, facilitating the accumulation of monocytes. CCL7 deficiency stabilizes monocyte distribution between the bloodstream and bone marrow, thereby regulating their migration from bone marrow to blood and from blood to inflamed tissues, ultimately producing an anti-inflammatory effect (Tsou et al., 2007). Additionally, CCL7 plays a crucial role in conditions such as osteoarthritis, where its secretion alleviates joint pain in murine models (Pandey et al., 2024).

Notably, key controversies and knowledge gaps persist in current research. The molecular mechanisms underlying CCL7-mediated macrophage plasticity remain unclear. Most studies only get results using single cell lines or mouse models, which fail to simulate the complex intercellular interaction microenvironment *in vivo*. Furthermore, when CCL7 is used to stimulate cells, the concentration applied often exceeds physiological levels (Kong et al., 2021). These limitations mean that research conclusions cannot fully reflect the true mechanisms under physiological or pathological conditions.

In addition, the immune systems of humans and mice have structural and functional differences. Consequently, it is unreasonable to directly extend the role of CCL7 in human diseases from findings conducted on mouse models. Currently, there are few clinical reports on CCL7 and most only observe changes in CCL7 expression, lacking verification of underlying mechanisms (Choi et al., 2004; Kurzejamska et al., 2019).

### Progression of fibrosis

Fibrosis, characterized by excessive extracellular matrix (ECM) deposition, is profoundly influenced by early-stage inflammation. Fibrotic processed are tightly associated with persistent early-stage inflammation. When the body gets injured, it sustains inflammatory responses that trigger the release of pro-inflammatory cytokines and growth factors, creating a pro-fibrotic microenvironment. These molecules not only enhance intercellular crosstalk between immune cells, stromal cells, and parenchymal cells but also activate the resident fibroblasts. A pro-fibrotic and anti-fibrotic balance governs the transition from acute inflammation to chronic fibrosis. Once this balance is disrupted, fibrosis progresses irreversibly in many cases.

As a proinflammatory chemokine, CCL7 is related to fibrosis progression in many tissues. In bleomycin-induced pulmonary fibrosis models, epithelial cell-derived CCL7 recruited macrophages by engaging CCR1/2/3 or CCR5/10, promoting their M1 polarization and driving fibrosis through activation of the NF-κB/STAT1 signaling pathway (Liu et al., 2023; Palomino & Marti, 2015; Zlotnik & Yoshie, 2012). *In vitro*, CCL7 directly stimulates dermal fibroblasts to express type I collagen (Ong et al., 2009; Ong et al., 2003). It activates TGF-β signaling, elevating the transcription of fibrogenic genes such as col1a2 and intensifying fibrotic progression. Furthermore, TGF-β stimulation enhances MCP-3 promoter activity within fibroblasts, suggesting a synergistic interplay that amplifies fibrogenic responses.

In tubulointerstitial fibrosis (TIF), marked by ECM accumulation within the interstitial space, leading to impaired renal function (Gonzalez et al., 2013; Klein et al., 2009). CCL7 exerts a dual role in the progression of TIF; it is detrimental in the early stage but beneficial in the late stage. In the early stage (Days 0–8), the fibrosis in CCL7-deficient mice was reduced, and infiltration of inflammatory cells and ECM production decreased. In contrast, during the late stage of obstruction (Days 10–14), TIF is exacerbated in CCL7-KO mice. Additionally, blocking the bradykinin B1 receptor to inhibit the expression of CCL7 can alleviate renal inflammation and fibrosis.

In addition, single-cell sequencing has revealed elevated CCL7 expression in the peripheral blood of patients with idiopathic pulmonary fibrosis (Unterman et al., 2024; Hasegawa, Takehara & Sato, 2006). Although CCL7 is strongly induced after acute kidney injury (AKI), CCL7 deficiency in mouse models fails to prevent the development of AKI and its progression to renal fibrosis (Casemayou et al., 2024). Therefore, CCL7 may not serve as a potential target for the prevention or treatment of AKI. Besides, the mechanisms underlying CCL7’s role in fibrosis also vary across different tissues. In conclusion, the switch of CCL7’s function in fibrosis depends on changes in the microenvironment.

### The role in obesity and glucose metabolism

Obesity and type 2 diabetes are characterized by chronic low-grade inflammation, which intensifies as elevated cytokine and chemokine levels promote immune cell infiltration into metabolic organs such as the liver, pancreas, and brain (Gregor & Hotamisligil, 2011). Within adipose tissue, the accumulation of immune cells fosters chronic inflammation and favors the polarization of macrophages toward the pro-inflammatory M1 phenotype. These macrophages, in turn, secrete additional pro-inflammatory mediators, including TNF-α and IL-1β, further exacerbating systemic metabolic dysregulation (Liu et al., 2020).

CCL7 expression increases in obesity mice and humans. Elevated CCL7 levels have been documented in obese mice compared to lean counterparts (Tsuhako et al., 2020). In high-fat diet-induced obesity models, CCL7 enhances the aggregation of inflammatory factors and PKA1 phosphorylation through CCR5 activation, while MMP9-mediated microenvironmental alterations contribute to the promotion of drug resistance and cellular invasiveness (Bou Malhab et al., 2024). In obese individuals, CCL7 expression is markedly upregulated in omental and subcutaneous adipose tissues relative to lean controls (Huber et al., 2008).

CCL7 regulates β-cell death and contributes to islet damage. In the mouse model of type 1 DM, T cell exosomes stimulate CCL7 expression in β cells to attract immune cells and exacerbate β cell death (Guay et al., 2019). Furthermore, Macrophages are recruited in adipose tissue during obesity, CCL7 may be associated with adipocyte inflammation and insulin resistance in type 2 DM (Inouye et al., 2007). However, the precise mechanisms by which CCL7 regulates obesity and modulates lipid and glucose metabolism remain to be fully elucidated.

## The Key Signaling Pathways of CCL7 Expression

**Figure 2:**
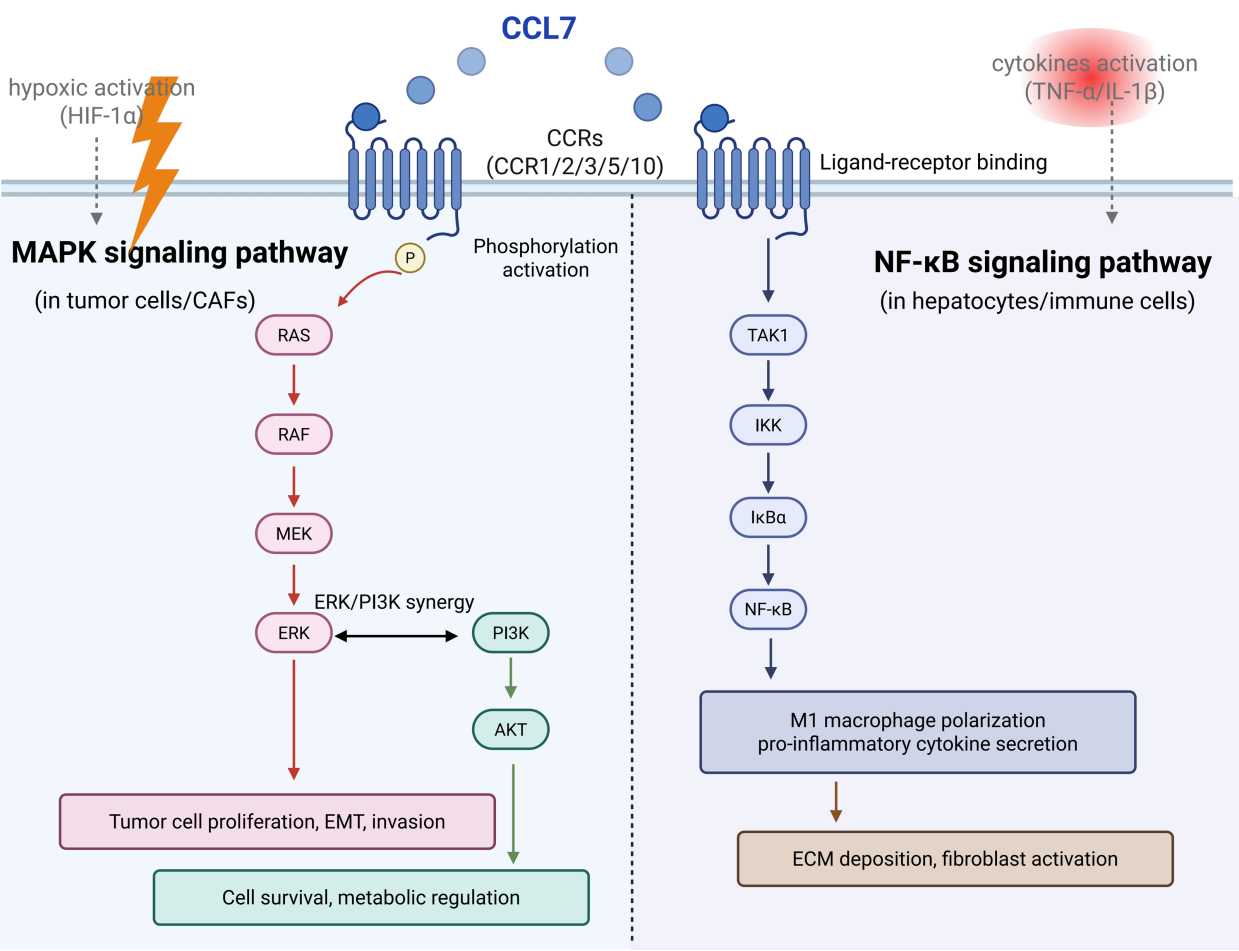
The key signaling pathways of CCL7 expression.

There are many molecular mechanisms and signaling pathways involved in the CCL7 regulation. CCL7 binds to CCRs and activates the downstream pathways. From the preview articles, it could influence the expression of JNK (Yang et al., 2022), ERK (Zhu et al., 2023), TNF-α (Zhu et al., 2023), and NF-κB (Xu et al., 2025). In total, NF-κB and MAPK pathways are reported to be frequently associated with inflammation and tumor development mediated by CCL7 (Fig. 2).

### NF-κB signaling pathway

The nuclear factor NF-κB pathway is a classical signaling pathway in immunity and inflammation (Sun, 2017). CCL7 is a kind of proinflammatory cytokine that could activate the NF-κB signaling pathway (Lawrence, 2009). CCL7 inhibition reduced macrophage and neutrophil influx into the lung to relieve allergic airways disease in mice, and it was associated with active NF-κB p65 and p50 levels (Girkin et al., 2015). In RAW cells, CCL7 switched macrophages to M1 polarization and blocking the receptor CCR1 reduced the activation of the NF-κB pathway by inhibiting the phosphorylation of IKK, IκBα and p65 expression (Xu et al., 2025). In a pulmonary fibrosis model, CCL7 drives M1 macrophage polarization *via* the NF-κB/STAT1 pathway, promoting ECM deposition (Zlotnik & Yoshie, 2012).

### MAPK signaling pathway

MAPK cascades are key signaling pathways that regulate cellular processes, including tumorigenesis, inflammation (Guo et al., 2020) and fibrosis. Among current articles, ERK prefers to be the common downstream of CCL7 in most diseases (Zhu et al., 2023; Chen et al., 2020a; Lee et al., 2016). CCL7 promoted human coronary artery smooth muscle cells proliferation by ERK1/2 and PI3K signaling pathway activation (Maddaluno et al., 2011). And CCL7 enhances colon cancer cell progression and EMT *via* CCL7-CCR3-ERK-JNK signaling (Lee et al., 2016).

### Microenvironmental cues regulate signaling pathways

Some cytokines promote NF-κB, while hypoxia promotes MAPK. In inflammatory disease, TNF-α/IL-1β activates NF-κB, leading to high CCL7 expression in CX3CR1hi macrophages and forming a pro-inflammatory loop (He et al., 2019). In colorectal cancer liver metastases, hypoxia upregulates CCL7 *via* HIF-1α, and the pathway shifts to ERK dominance, enhancing tumor invasiveness and drug resistance (Chang et al., 2024; Mohr et al., 2017).

### Different cell source affects pathway selection

In CAFs, CCL7 enhances tumor progression, and inhibiting NF-κB significantly reduces its pro-tumor effects (Jung et al., 2010). But hepatocytes upregulate CCL7 expression *via* NF-κB, recruiting inflammatory cells to exacerbate damage (Kong et al., 2021). Colorectal cancer cells could secrete CCL7 to activate the MAPK pathway to drive proliferation (Lee et al., 2016). In coronary artery smooth muscle cells, CCL7 synergistically promotes cell proliferation *via* the ERK and PI3K pathways (Chen et al., 2020a).

In total, NF-κB dominates in inflammation/fibrosis, while MAPK dominates in tumor metastasis. The differences between the two pathways are the molecular basis for the multifunctionality of CCL7.

## Clinical and Translational Perspective

Given the multifaceted roles of CCL7 in tumors, inflammatory diseases, fibrosis, and metabolic disorders, there is potential for CCL7 to be a biomarker and therapeutic target.

### CCL7 as a diagnostic and prognostic biomarker

CCL7 expression correlates with disease severity, progression, and treatment response across multiple pathological contexts, supporting its utility as a clinical biomarker. In ovarian cancer, CCL7 deficiency is associated with reduced sensitivity to PD-1 inhibitors, further validating CCL7 as a predictive biomarker for immunotherapy response (Mollaoglu et al., 2024). In TIF, urinary CCL7 levels are significantly higher in patients with early-stage renal injury and decrease with effective anti-fibrotic treatment, suggesting its potential as a non-invasive diagnostic marker for early TIF (Gonzalez et al., 2013). In type 1 diabetes (T1D), CCL7 expression in pancreatic islets is associated with β-cell death and T cell infiltration (Guay et al., 2019).

### Therapeutic strategies targeting CCL7

Targeting CCL7 with multiple approaches has the potential to utilize its anti-disease functions. Evasins could bind chemokines and block their interaction with receptors. Evasin Class A1 and Class A3 exhibit high affinity for CC chemokines, including CCL7. Small-molecule inhibitors or neutralizing antibodies or gene knockout approaches targeting CCL7’s receptors (CCR1, CCR2, CCR3) inhibit further progression of the disease in preclinical studies (Lee et al., 2016; Schmall Al-Tamari et al., 2015; Xu et al., 2025). Combining CCL7-targeted agents with existing treatments enhances therapeutic efficacy. In NSCLC, CCL7 overexpression synergizes with anti-PD-1 therapy by recruiting cDC1 and expanding CD8^+^ T cells (Zhang et al., 2020).

## Conclusion and Prospect

In summary, CCL7 plays a pivotal role in tumorigenesis, inflammation, and systemic homeostasis, mainly through its capacity to modulate monocyte responses. By binding to its receptors, CCL7 facilitates the infiltration of inflammatory cells and can exert both pro- and anti-inflammatory effects. In the early stages of the disease, CCL7-mediated macrophage recruitment contributes to pain relief, anti-inflammatory activity, and the maintenance of both TME and systemic stability. However, as inflammation progresses and macrophage phenotypes shift, systemic homeostasis deteriorates, fibrosis worsens, and tumor growth and metastasis are promoted.

Regarding tumor progression, CCL7 shares 71% sequence homology with the extensively studied chemokine CCL2, which has well-documented roles in tumorigenesis and EMT(Chen et al., 2022; Pozzi & Satchi-Fainaro, 2024). In contrast, the tumor-promoting mechanisms of CCL7 remain less well-defined. Current evidence shows that CCL7/CCR1 antagonists can suppress colorectal cancer bone metastasis and that combined IL-4 blockade and PD-1 inhibition can reverse CCL7-mediated immunosuppression in ovarian cancer (Mollaoglu et al., 2024). Elucidating additional signaling pathways related to CCL7 will undoubtedly enhance future clinical interventions.

The immunomodulatory potential of CCL7 is also gaining recognition, particularly in its ability to direct macrophages and other immune cells to target tissues. In an HDM-induced asthma mouse model, elevated CCL7 expression by B cells recruits macrophages to the lungs, exacerbating pulmonary inflammation (Waldstein et al., 2024). In DSS-induced colitis models, serum levels of CCL7 are significantly increased, accompanied by NF-κB pathway activation (Cho et al., 2015). Since CCL7 primarily exerts its immunomodulatory effects through binding to structurally homologous G protein-coupled receptors (CCRs), genetic manipulation or pharmacological blockade of CCRs has been shown to attenuate liver injury in animal models (Choi et al., 2015; Proost, Wuyts & Van Damme, 1996).

Existing evidence underlines the therapeutic potential of targeting CCL7. However, the translation of these findings into clinical settings remains hindered. In the future, we should develop CCL7-targeted inhibitors and improve antibody-drug conjugates with high affinity for CCL7. We still need more prospective trials to confirm serum CCL7 levels as diagnostic markers for inflammation, fibrosis or diabetes. Also, monotherapies targeting CCL7 often show limited efficacy due to redundant signaling pathways. Future studies should investigate synergistic combinations.

Despite these advances, the precise mechanisms of CCL7 remain to be fully elucidated. While its upregulation and the associated accumulation of macrophages and B cells are well-documented across various diseases, critical questions remain regarding the cellular sources of CCL7, upstream regulators of its expression, and the specific effects of CCL7-CCR interactions in different pathological contexts. Addressing these gaps holds significant scientific promise for advancing our understanding of the immune system and related disease processes.

## Additional Information and Declarations

### Data Availability

The following information was supplied regarding data availability: This is a literature review.
